# Elucidating the molecular landscape of the stratum corneum

**DOI:** 10.1073/pnas.2114380119

**Published:** 2022-03-17

**Authors:** Nichola J. Starr, Mohammed H. Khan, Max K. Edney, Gustavo F. Trindade, Stefanie Kern, Alexander Pirkl, Matthias Kleine-Boymann, Christopher Elms, Mark M. O'Mahony, Mike Bell, Morgan R. Alexander, David J. Scurr

**Affiliations:** ^a^School of Pharmacy, University of Nottingham, University Park, Nottingham NG7 2RD, United Kingdom;; ^b^Department of Chemical and Environmental Engineering, University of Nottingham, University Park, Nottingham NG7 2RD, United Kingdom;; ^c^National Centre of Excellence in Mass Spectrometry Imaging, National Physical Laboratory, Teddington, Middlesex TW11 0LW, United Kingdom;; ^d^Research and Development, IONTOF GmbH, 48149 Münster, Germany;; ^e^No7 Beauty Company, Walgreens Boots Alliance, Nottingham NG90 1BS, United Kingdom

**Keywords:** skin, mass spectrometry, 3D OrbiSIMS, stratum corneum

## Abstract

Characterization of the molecular structure of skin, especially the barrier layer, the stratum corneum, is a key research priority for generating understanding to improve diagnostics, aid pharmaceutical delivery, and prevent environmental damage. Our study uses the recently developed 3D OrbiSIMS technique to conduct in situ analysis of ex vivo human skin tissue and reveals the molecular chemistry of skin in unprecedented detail, as a result of the step change in high mass resolving power compared with previous studies. This characterization exposes the nonhomogeneity of the stratum corneum, both laterally and as a function of depth. Chemical variations relating to fundamental biological processes, such as the epidermal cholesterol sulfate cycle, are visualized using in situ analysis. We are able to resolve the debate around the chemical gradients present within the epidermis, for example, whether palmitic acid is of sebaceous origin or a true component of the stratum corneum. Through in situ depth analysis of cryogenically preserved samples, we are able to propose that it is actually a component of both surface sebum and the intrinsic lipid matrix. This approach also suggests similarity between the epidermis compounds found in human and porcine skin as a function of depth. Since porcine skin is a widely used model for permeation testing this result has clinical relevance. In addition to using this technique for endogenous species, we have used it to demonstrate the permeation of a commercially important antiaging peptide into the human stratum corneum. Due to its chemical similarity to native skin components and exceptionally low effective concentration, this information was previously unattainable.

## Significance

Skin is recognized as an intricate assembly of molecular components, which facilitate cell signaling, metabolism, and protein synthesis mechanisms in order to offer protection, regulation, and sensation to the body. Our study takes significant steps to characterize in more detail the complex chemistry of the skin, in particular by generating a better understanding of the uppermost layer, the stratum corneum. Using a state-of-the-art 3D OrbiSIMS technique, we were able to observe the depth distribution, in situ, for a wide range of molecular species. This unprecedented molecular characterization of skin provides information that has the potential to benefit research into fundamental processes, such as those associated with skin aging and disease, and the development and delivery of effective topical formulations.

The skin is described using its three distinct layers, namely, the hypodermis, dermis, and epidermis. The outermost layer, the epidermis, is chemically complex and contains five sublayers, defined by the differentiation stage of keratinocyte cells. The uppermost sublayer, the stratum corneum (SC), is the body’s primary defense against the environment, despite an average thickness of only 20 cell layers (1). This protective role includes the regulation of water and heat loss, resistance against mechanical stress, the prevention of xenobiotic permeation, and protection against ultraviolet (UV) exposure. Once thought to act as a physical barrier of dead corneocyte cells, it is now recognized that the surrounding lipid matrix acts as a sophisticated biosensor, regulating metabolic activity in response to external stimuli (2–4). This lipid matrix is unique among biological membranes, both in terms of composition and organization, and it is known that these lipids act as bioactive compounds in numerous cellular processes. However, the mechanisms behind these regulatory processes and the involvement of individual lipid species are still not fully understood (4). Improving the molecular characterization of the SC is therefore at the forefront of current skin research to provide the basis to enable better diagnostics, improve pharmaceutical delivery, and enable strategies to reduce environmental damage.

It is widely acknowledged that the main lipid classes present in the SC matrix are ceramides, free fatty acids and cholesterol, cholesterol sulfate, and cholesterol esters (1). Unlike the underlying epidermal layers, the SC contains almost no phospholipids, as their depletion accompanies the differentiation of the keratinocyte cells, known as cornification, which creates the skin’s physical barrier. Although the main components are known, the exact composition of this lipid matrix is continually being updated through ongoing research. To date, 17 subclasses of ceramides have been recognized in human SC (5), with Laffet et al. recently demonstrating the identification of 1,000 different ceramide molecules from human and porcine SC (6). Free fatty acids exist as both saturated and unsaturated compounds, with 30 different species currently detected with chain lengths ranging from 14 to 34 carbon atoms. Of these, lignoceric and hexacosanoic acid (C24:0 and C26:0) have been demonstrated to be the most abundant (7, 8). In addition to this complex mixture, the SC is also populated with compounds relating to both surface sebum and a mix of hygroscopic species termed natural moisturizing factor (NMF). These compounds play a key role in the hydration of the skin (9, 10). Sebum, secreted onto the surface through sebaceous glands, is known to contain triglycerides, wax esters, free fatty acids, and squalene, with smaller quantities of cholesterol and cholesterol esters also present (11, 12). Enzymatic degradation of the binding protein filaggrin results in SC localization of free amino acids, notably histidine, glutamine, arginine, and their derivatives; urocanic acid; pyroglutamic acid or 2-pyrrolidone-5-carboxylic acid (PCA); and urea/ornithine (13, 14).

Given the complex nature of the SC, it is unsurprising that imbalances within this chemical matrix have been associated with numerous biological changes, including aging (15–17), UV damage (15, 17–19), and multiple skin disorders (3, 20–23). Different analytical techniques have been employed to investigate the SC, most notably chromatographic mass spectrometry (8, 22, 24). However, although this technique enables the accurate identification and quantification of biological molecules, it relies on the physical separation of individual skin layers, followed by successful extraction of the compounds of interest. Not only is this time consuming but also much information regarding the distribution of key species within the SC, both laterally and as a function of depth, is lost. This means that, for most analytical studies, the chemical composition of the SC is assumed to be homogenous. The use of tape stripping as a sampling method, removing individual corneocyte layers for analysis, can be used to circumvent this issue. Yet, this method of sampling can be subject to large variabilities and requires the strict control of multiple parameters (25). Limited instrument sensitivity can also dictate that strips need to be pooled for analysis, causing a loss of depth resolution.

Time-of-flight secondary ion mass spectrometry (ToF-SIMS) offers an advantage over other techniques, as it can provide in situ analysis with minimal sample preparation, while still offering high chemical sensitivity and specificity. Another unique feature of this technique is its ability to probe compound distribution both laterally and as a function of sample depth, through either surface or depth profile analysis modes down to a microscopic scale for molecular characterization. It is therefore becoming increasingly popular for skin tissue analysis, with numerous groups demonstrating the effectiveness of this technique to analyze both native and exogenously altered skin samples, including an analysis of tape-stripped SC, cross sections, and ex vivo full-thickness skin (15, 26–32).

A small number of analytical studies, using various instrumentation, have demonstrated a change in lipid composition with epidermal depth (15, 27, 33–35), suggesting that the assumption that the lipid matrix is homogeneous is an oversimplification. However, all of these suffer from limited depth resolution or lipid specificity. The most comprehensive analysis carried out using ToF-SIMS was by Sjövall et al. (29) in 2018, who imaged a cross-sectioned skin sample to reveal the two-dimensional (2D) spatial distribution of a variety of molecular species and highlight their localization within the main skin layers. However, due to limitations with the ToF-SIMS instrumentation, the presence of subtle chemical gradients, in particular within individual skin layers, remains unexplored.

In order to address the lack of insight into the three-dimensional (3D) molecular landscape of the SC, we have exploited the improved capabilities of the recently developed 3D OrbiSIMS profiling technique using a Hybrid SIMS instrument (IONTOF GmbH) (36). This instrument combines traditional ToF-SIMS with Orbitrap mass spectrometry and as a result can offer significant analytical advancements for biological analysis compared with previous ToF-SIMS instruments. The exceptional mass resolution (>240,000) and mass accuracy (<1 ppm) enable unique biological molecule identification of large diagnostic molecular fragments, generated by a low-energy argon gas cluster primary ion beam. In addition, the ability to conduct this analysis on samples maintained in a cryogenic state is hugely important to preserve the native state and improve the detection of biomolecules (37, 38). The in situ analysis employed in SIMS, combined with gas cluster etching to generate depth profile analysis, is ideally suited to 3D characterization of skin and has allowed us to delineate the chemical composition of human SC, revealing previously unreported trends in the chemistry of this important sensory skin barrier.

## Results and Discussion

### Identifying the Importance of Cryogenic Sample Fixation for Skin Analysis.

**Fig. 1.**
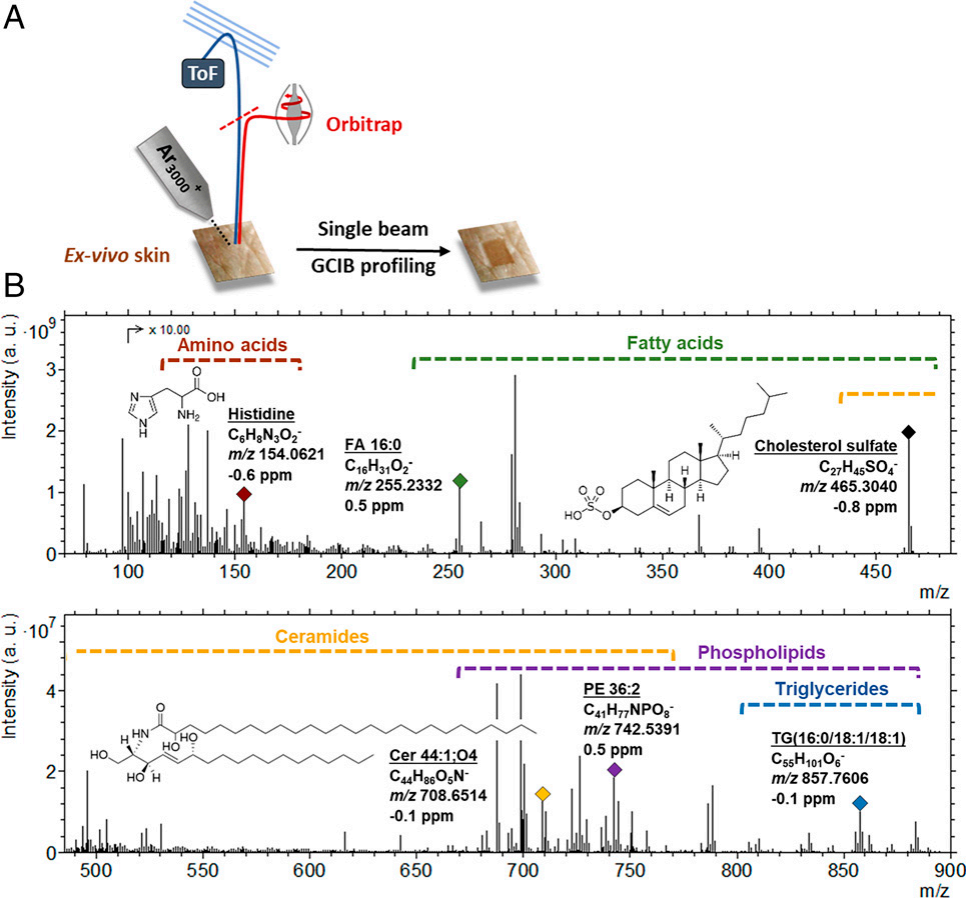
3D OrbiSIMS depth profile analysis of frozen hydrated ex vivo human skin tissue. (*A*) A schematic illustrating the process. (*B*) An example negative polarity spectrum. The spectrum presents the total ion intensity for the 250 × 250 µm area summed across the whole depth profile (total applied ion dose is 2.88 × 10^13^). Using the SIMS-MFP software, we were able to putatively assign over 140 different species across 5 compound classes (amino acids, fatty acids, ceramides, phospholipids, and triglycerides). The mass regions for these classes are highlighted on the spectrum, and an example compound is labeled for each class, detailing the molecular assignment, chemical formula, and the observed mass (*m/z*) and deviation. a.u., arbitrary units; FA, fatty acid; Cer, ceramides; TG, triglycerides; PE, phosphatidylethanolamine.

Ex vivo full-thickness human skin tissue samples were primarily used for this study, and a single gas cluster ion beam was employed to both sputter through the skin and generate secondary ions, which were analyzed using an Orbitrap analyzer to generate a depth profile (Fig. 1*A*). As part of the initial method development, ex vivo full-thickness porcine skin samples were analyzed in either a dehydrated state at room temperature (RT) or frozen (−170 °C) to maintain the native hydration state of the sample (termed “frozen hydrated”). There was a significant intensity reduction for the majority of ions generated from the dehydrated sample, especially the higher mass species (*SI Appendix*, Fig. S1*A*), with some ions absent from the spectra altogether (examples in *SI Appendix*, Fig. S1*B*). This is consistent with previous studies that postulate an ionization enhancement due to the presence of water (37–40). Cryofixation of samples for SIMS analysis also has the advantage of preventing lipid migration and maintaining native structure. The frozen hydrated sample preparation method is therefore used subsequently.

### Elucidating SC Molecular Gradients.

Depth profiling was conducted on frozen hydrated ex vivo human skin samples in both negative and positive polarity with more than 8,000 ions detected with an intensity above 1 × 10^5^ in the former and more than 1,000 ions in the latter. Secondary ion mass spectra consist of both ionized molecular species with very little modification, such as gain or loss of a proton, and more fragmented ions. An example negative polarity 3D OrbiSIMS spectrum is shown in Fig. 1*B*. In order to assign structures to the large number of detected ions and delineate the chemical complexity of this tissue, the recently developed chemical filtering (SIMS-MFP) method was used (41). This predicts the molecular formula of species in a 3D OrbiSIMS dataset based on accurate mass determination of an elemental formula and categorizes the species based on their double bond equivalents. This enables targeted searches to be carried out for different compound classes based on unique functional groups contained within. For the purpose of our study, we focus on the negative polarity data, due the large number of species present. These data are taken from one analysis area, and yet it was repeatable across the four different areas analyzed. The following molecular compositions were used to focus the software search, in order to focus on species known to be present within the SC and underlying epidermis: C_n_H_n_O_2_ (fatty acids, wax esters, cholesterol esters), C_n_H_n_O_6_ (triglycerides), C_n_H_n_O_n_N_1_ (ceramides, amino acids, and derivatives), C_n_H_n_O_n_N_n_P_1_ (phospholipids), and C_n_H_n_S_1_O_n_ (sulfate-based lipids). The assignments were then verified by comparison against the LIPID MAPS Structure Database (42) and/or previous literature, allowing us to putatively annotate over 140 molecular species across 5 different compound classes (Fig. 1*B*).

**Fig. 2.**
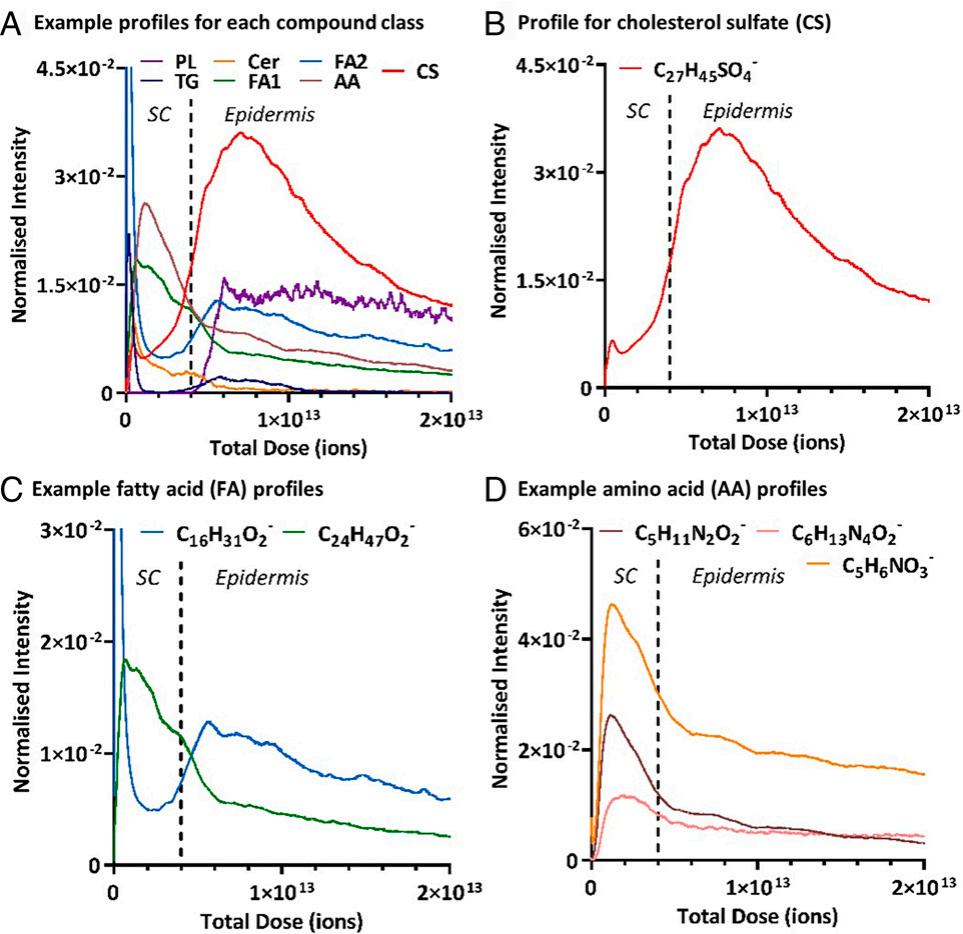
3D OrbiSIMS negative polarity depth profile data showing the ion intensity variation as a function of ion dose/skin depth for various putatively assigned compounds. The primary ion beam was Ar_3000_^+^. The ion intensities have been normalized to the total ion count, and the profiles have been compressed using a running average method (using a 100 data point average). The SC*–*epidermal boundary, approximated with a dotted vertical line, has been determined based on the ion intensity variation of the phospholipid species (*SI Appendix*, Fig. S2). (*A*) Example ions from each identified compound class as follows: PL (phospholipids; C_41_H_77_NPO_8_^−^), TG (C_55_H_101_O_6_^−^), Cer (C_44_H_86_NO_5_^−^), FA1 and FA2 (FAs 1 and 2; C_27_H_47_O_2_^−^ and C_16_H_31_O_2_^−^), AA (amino acids; C_5_H_11_N_2_O_2_^−^), and CS (cholesterol sulfate; C_27_H_45_SO_4_^−^). The normalized intensity for the phospholipid ion has been multiplied by a factor of 50. (*B*) Cholesterol sulfate molecular ion (C_27_H_45_SO_4_^−^). (*C*) FAs, palmitic acid (C_16_H_31_O_2_^−^), and lignoceric acid (C_24_H_47_O_2_^−^). (*D*) NMF compounds, the amino acid arginine (C_6_H_13_N_4_O_2_^−^) and amino acid derivatives PCA (C_5_H_6_NO_3_^−^) and ornithine (C_5_H_11_N_2_O_2_^−^). The normalized intensity for the arginine ion has been multiplied by a factor of 10.

Examining the depth profiles for these assignments revealed that these different compound classes exhibited different trends with depth away from the surface of the skin, with some classes even showing a variation between individual species. The negative polarity depth profile data for an example ion from each detected compound class, specifically, triglycerides, ceramides, fatty acids, phospholipids, amino acids and their derivatives and sulfated compounds are shown in Fig. 2*A*. The full list of identified species and their respective depth profiles, separated by class, can be found in the supplementary information, *SI Appendix*, Tables S1–S5 and *SI Appendix*, Figs. S2–S6, respectively. The ion intensity, representing the relevant concentration of a molecule, is plotted as a function of total ion dose, which equates to the depth below the surface of the skin. Due to the nonhomogeneity of the SC thickness across the 250 × 250 µm area analyzed, distinct ion intensity changes are not expected and gradual molecular gradients are observed in comparison with a sample with a flat interface. However, our study demonstrates that different molecular species demonstrate differently sloped gradients, which suggests that these are real molecular variations with depth and not artifacts of a heterogeneous SC layer.

This heterogeneity, plus the impracticalities of physically measuring the SC depth for every individual analysis area, resulted in a method to intrinsically monitor the location within the SC in situ while profiling (26). Specifically, the intensity of a phospholipid ion was monitored throughout the profile, as phospholipids are at a minimal level within the SC until the transition into the underlying epidermis. This intensity increase is clearly observed in all the phospholipid species that we identified (*SI Appendix*, Fig. S2), enabling the SC–epidermal boundary to be easily identified as a function of skin depth. In contrast, both triglycerides and ceramides were shown to be present within the SC but exhibited different trends to each other. Triglyceride species showed a high ion intensity at the surface of the skin which rapidly decreased to a baseline level in the latter part of the SC, which agrees with their known skin sebum origins. However, the depth profiles (*SI Appendix*, Fig. S3) highlight that these species are also present, to a much lesser extent, in the upper part of the epidermis. Ceramides on the other hand are solely localized to the SC, as previously believed, reaching a baseline level once the SC–epidermal boundary has been passed. Yet the profiles for these species (*SI Appendix*, Fig. S4) highlight that this localization is not uniform across the SC layer, with the ion intensity decreasing initially from the skin surface before reaching a maximum in the upper-central part of the SC. These observed molecular gradients exemplify the usefulness of this in situ depth profiling approach. Although, it is currently understood that ceramides are only found within the SC (1, 29), demonstrating their localization to the central portion has not previously been achievable.

Although cholesterol sulfate is a minor component of the SC lipid matrix in terms of concentration, it plays an essential key role in desquamation and skin regeneration, with increased levels linked to both aging and skin disorders (15, 43, 44). The “epidermal cholesterol sulfate cycle” was first hypothesized in 1984 by Epstein et al. (45) and describes fluctuating levels of this molecule throughout the epidermis, increasing from the basal layer to reach a peak in the stratum granulosum, before decreasing once the SC is reached. Quantification of cholesterol sulfate levels in extracted skin layers has been achieved through chromatographic mass spectrometry (43), but to date, this concentration fluctuation has not been visualized in situ. Recent work by Sjövall et al. (29) used ToF-SIMS analysis of ex vivo human skin cross-sections to illustrate a decrease in cholesterol sulfate intensity in the SC region compared with the underlying epidermis. The depth profile for the cholesterol sulfate molecular ion (C_27_H_45_SO_4_^−^) observed in this study (Fig. 2*B*) not only confirms this trend but also offers a more detailed insight into the distribution gradient of this molecule within individual layers. It is apparent that cholesterol sulfate steadily increases throughout the SC but then reaches a maximum concentration in the upper portion of the underlying epidermis, which based on its location is assumed to correlate to the stratum granulosum, before then starting to decline. This agrees with the proposed relative levels of cholesterol sulfate and the mechanisms behind the epidermal cholesterol sulfate cycle, which has previously only been shown through a layer vs. layer comparison. This trend is shown repeatedly for the three different human donor samples analyzed (*SI Appendix*, Fig. S7). The use of the in situ 3D OrbiSIMS profiling method has enabled the subtlety of this continuous cholesterol sulfate gradient to be observed as a function of skin depth.

Free fatty acids are a major lipid class within the SC. Not only is the 3D OrbiSIMS method able to uniquely identify individual fatty acid species with mass deviations of <0.9 ppm, but also it revealed differing depth distributions within the skin for individual species within this class (*SI Appendix*, Fig. S5). The intensity profiles for two example fatty acids, namely, palmitic and lignoceric acid, are shown in Fig. 2*C*. Palmitic acid (C16:0) exhibits an initial rapid decrease in molecular ion (C_16_H_31_O_2_^−^) intensity, reaching a baseline level in the upper part of the SC. However, toward the deeper part of the SC, the intensity for this molecule starts to increase again, achieving a local maximum intensity as the underlying epidermis is reached. This fatty acid has previously been shown to be present within the SC, but there is debate over whether it is sebum related or originates from the intrinsic lipid matrix (46). The results seen here suggest that palmitic acid comes from both sources. It appears to be present on the very surface of the skin before its rapid decrease in intensity, behavior which would be expected from sebum components, but is also present to a lesser extent within the lipid matrix of the lower part of the SC. In contrast to palmitic acid, the molecular ion (C_24_H_47_O_2_^−^) intensity of lignoceric acid (C24:0) experiences an initial increase once the surface of the skin has been passed. This is followed by a gradual decline in intensity, reaching a baseline level upon entering the underlying epidermis. This trend is consistent with previous studies highlighting its localization solely within the SC layer, as one of the most abundant fatty acids present in the lipid matrix (7, 8). However, the profiling method used here suggests that, although localized to the SC, lignoceric acid is not uniform across this layer. It is almost devoid at the skin surface and then present to a greater extent within the upper layers compared with the lower layers (Fig. 2*C*). These trends are reproduced in the three human donor samples analyzed with a reduced relative intensity of lignoceric acid observed in donor 2 (*SI Appendix*, Fig. S7*B*). It is known that fatty acids are present within the SC as free species but also as components of larger species, such as ceramides. Without a more comprehensive analysis, we cannot say conclusively that the ions we are observing originate from the free form of this species. However, as the fatty acids present a different depth profile trend compared with the larger lipid species, it suggests that they are likely to be free fatty acid molecules rather than fragment ions.

In addition to the major lipid components, the SC also contains NMF, a mix of hygroscopic molecules including amino acids and their derivatives, some of which we were able to identify and show their SC localization (*SI Appendix*, Table S5 and Fig. S6). These compounds are a product of the enzymatic breakdown of filaggrin, and although they were previously demonstrated to be present within the SC, little information is known about their depth distribution. A recent paper by Kubo et al. (27) focused on the localization of one of these amino acids, namely, arginine, through ToF-SIMS ion image analysis of cross-sectioned mouse tail. Their findings suggested that the SC was made up of three distinct layers, with arginine present to a greater extent in the upper and central portions of the SC. Our study was able to accurately identify secondary ions relating to not only arginine but also the other main NMF components. The molecular ion intensity profiles of three of these compounds, namely, arginine (C_6_H_13_N_4_O_2_^−^), its derivative ornithine (C_5_H_11_N_2_O_2_^−^), and the most abundant NMF molecule PCA (C_5_H_6_NO_3_^−^) (13, 14), are highlighted in Fig. 2*D*. It is apparent that they are all localized within the SC, as expected (shown for the three donor samples analyzed in *SI Appendix*, Fig. S7), and all follow the same intensity trend with depth. Specifically, the trend is an absence at the immediate surface, before reaching a maximum intensity within the upper portion of the SC, followed by a constant decrease in intensity as the underlying epidermis is approached. This result confirms the outcome published by Kubo et al. (27), with greater levels of these molecules present in the upper and central part of the SC. However, this profiling method provides the ability to assess the relative quantities of these molecules as a constantly changing gradient throughout the SC, rather than the three distinct layers portrayed in the ion images presented by Kubo et al. (27).

It is important to note that in addition to the epidermal lipids, we were also able to detect and observe trends as a function of skin depth for peptide sequences characteristic to three key skin proteins, namely, corneodesmosin, keratin, and collagen. Protein analysis of skin using cryo-OrbiSIMS has been previously demonstrated by Kotowska et al. (47), but a detailed investigation into these trends is outside the scope of this current study.

Overall, the 3D OrbiSIMS profiling method, coupled with the chemical filtering (SIMS-MFP) analytical approach, has enabled an extensive analysis of the chemical species present within the upper layers of the skin, revealing previously unexplored gradients with skin depth for various compound classes. This has been achieved by successfully combining the chemical specificity usually associated with traditional mass spectrometry techniques with the localization information produced from in situ SIMS analysis. Positive polarity data were also assessed and showed a similar series of trends and behaviors denoting the same species as observed in the negative dataset within different layers of the skin. As with the negative polarity data within similar chemical species, both similar and dissimilar trends were observed (*SI Appendix*, Fig. S8). A more comprehensive list of ceramides, fatty acids, and amino acid secondary ions observed in the positive datasets are shown in *SI Appendix*, Tables S6–S8.

The general trends discussed in skin chemistry from human samples were also observed within porcine skin tissue analyzed with a comparison of the data shown in *SI Appendix*, Fig. S9. Porcine skin is routinely used as an alternative to human skin for pharmaceutical and clinical testing, as it has been demonstrated to be similar in both structure and chemical composition to human skin (48, 49). Our data would also suggest that there is a significant similarity extending to the subtle chemical gradients we can now identify using the 3D OrbiSIMS method.

### Exogenous Species within Native Tissue.

From the native ex vivo human skin analysis, we also detected the exogenous species sodium lauryl sulfate (SLS), a popular anionic surfactant used in many hygiene products. The depth profile for this compound, identified from the [M-Na]^−^ ion (C_12_H_25_SO_4_^−^) (*SI Appendix*, Fig. S10) also highlights a localization to the SC. However, it is apparent that this molecule does not decrease to zero intensity once the skin surface has been passed, as is observed with sebum-related native species like palmitic acid (Fig. 2*C*). Instead, it shows an initial increase in the upper layers of the SC, before declining rapidly, and then at a lower rate. This suggests that this exogenous compound, not designed to intentionally penetrate the skin, is not solely present on the surface but has actually permeated into the SC as a result of exposure to SLS-containing products.

### Lateral Distribution of SC Compounds.

**Fig. 3.**
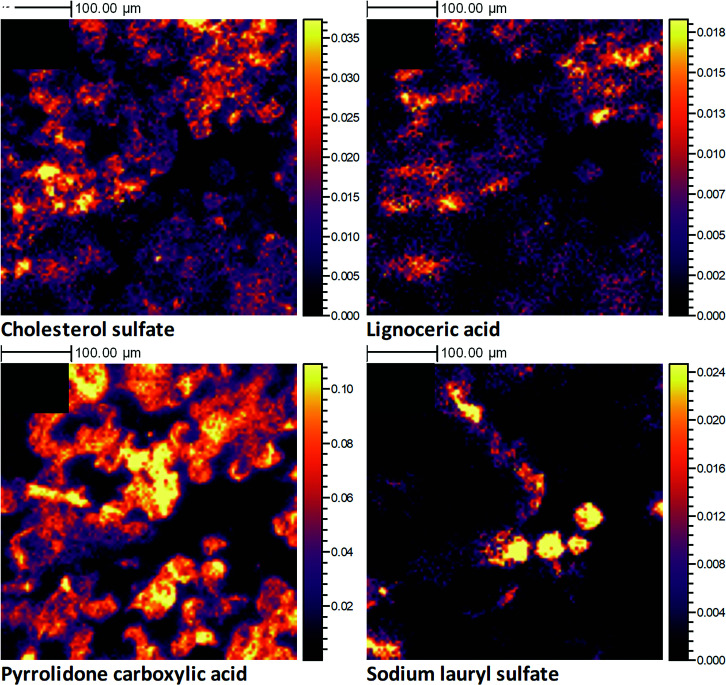
3D OrbiSIMS high-resolution negative polarity ion images, produced from the analysis of an individual human corneocyte skin layer, collected in vivo using a tape-stripping method. The primary ion beam was Ar_1700_^+^. The data shown are from tape strip 7, which represents the middle of the SC. The ion images illustrate the 2D spatial distribution for the following molecular ions: cholesterol sulfate (C_27_H_45_SO_4_^−^) (*A*), lignoceric acid (C_24_H_47_O_2_^−^) (*B*), pyrrolidone carboxylic acid (C_5_H_6_NO_3_^−^) (*C*), and SLS (C_12_H_25_SO_4_^−^) (*D*). All ion images have been normalized to the total ion image and their relative intensity scales set to 50% of the maximum.

Although the 3D OrbiSIMS single-beam profiling method applied here can provide useful insights into the chemistry of the SC as a function of depth, it is not able to offer information regarding lateral composition. A combination of the in vivo tape-stripping sampling method with 3D OrbiSIMS imaging was therefore used to investigate the lateral distribution of SC compounds within an individual corneocyte layer. Molecular ion images for four example compounds found in the SC, specifically cholesterol sulfate, lignoceric acid, PCA, and the exogenous SLS, are displayed in Fig. 3, following an analysis of a tape strip sample proposed to represent the middle of the SC layer (tape strip number 7). These compounds displayed divergent depth distributions, and from these ion images, it is clearly apparent that they also present different lateral distributions within a single SC layer. This further confirms that the SC is not a homogenous layer, as is regularly portrayed, but in fact a complex mixture of compounds that vary in concentration both within and between layers. Although cholesterol sulfate (Fig. 3*A*), lignoceric acid (Fig. 3*B*), and PCA (Fig. 3*C*) all display a similar level of coverage across the imaged area, there are clear areas of localization that differ between the compounds. We were even able to demonstrate that the lateral localization varied between different NMF compounds (*SI Appendix*, Fig. S11), indicating that the breakdown of these amino acids into their derivatives is not a uniform process within a layer. The depth profile for SLS (*SI Appendix*, Fig. S9) highlighted the permeation of this molecule into the SC, which is confirmed by a clear presence of this molecule within this sampled corneocyte layer (Fig. 3*B*). However, it appears that this permeation is not uniform across the SC layer, instead localizing in small patches of corneocytes within the imaged area. This example dataset confirms that the method can be utilized to study the localization of both native and exogenous species within a single corneocyte layer, even within individual corneocyte cells. It should be noted that the primary ion dose applied for this analysis (even for a single image scan) exceeds the static SIMS regime and as such the data are not surface specific but actually represent the chemistry from both the surface and subsurface of these individual corneocytes. The extent of sample etching resulting from the total of 4 imaging scans acquired is demonstrated in the pre- and postoptical images (shown *SI Appendix*, Fig. S11 *A* and *B*, respectively) where a significant reduction in the visible corneocytes is observed postanalysis within the analytical crater region.

### Peptide Delivery through the SC.

In addition to the endogenous species discussed, we have demonstrated the ability to detect and identify exogenous molecules present in human skin following topical application of compounds.

**Fig. 4.**
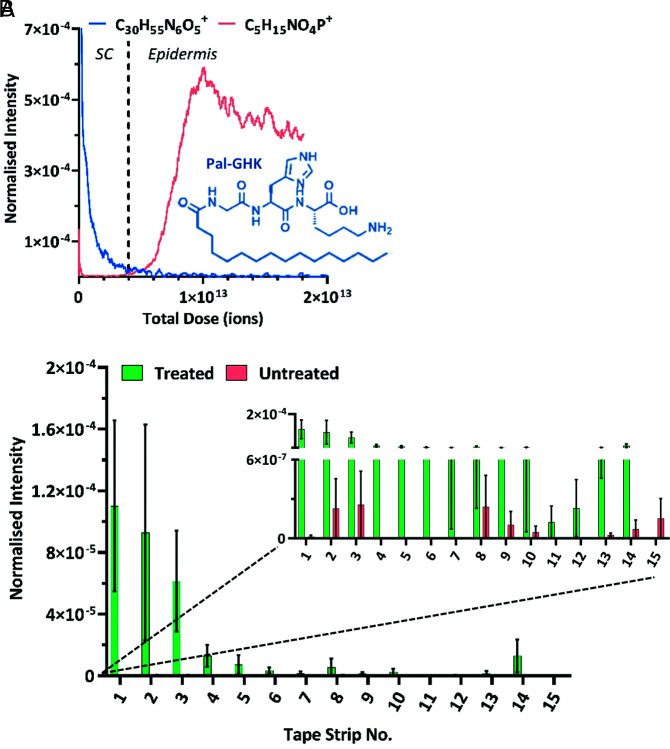
3D OrbiSIMS data showing the ion intensity variation as function of skin depth for various compounds, highlighting the subtle changes in skin chemistry that can be detected using this method. The primary ion beam was Ar_3000_^+^.The ion intensities have been normalized to the total ion count. (*A*) Data demonstrating the permeation of an exogenous compound, the palmitoyl oligopeptide Pal-GHK (C_30_H_55_N_6_O_5_^+^), into the SC layer of ex vivo human skin tissue. The SC*–*epidermal boundary has been determined and approximated with a dotted vertical line, based on the ion intensity variation of the intrinsic phosphocholine molecular ion (C_5_H_15_NO_4_P^+^). The profiles have been compressed using a running average method (100 data points) and the skin depth is represented by total ion dose. (*B*) Data showing the normalized intensity for the Pal-GHK molecular ion (C_30_H_55_N_6_O_5_^+^) as a function of tape strip number, produced from 3D OrbiSIMS analysis of human tape strips collected in vivo following application of a commercially relevant formulation. The graph contains data from 15 sequential tape strips collected from both treated and untreated sites and is represented as the average and SE of 3 different volunteers (*n* = 3).

Palmitoyl tripeptide-1 (Pal-GHK) is a synthetic palmitoyl oligopeptide that is highly utilized in the antiaging cosmetic market (50). Commercial formulations containing the peptide have been shown to have clear antiaging benefits (51, 52), stimulating the deposition of fibrillin-1 microfibrils and reducing the facial wrinkle clinical grade (51). Using the 3D OrbiSIMS profiling method, we were able to track the permeation of this peptide through ex vivo human skin at commercially relevant levels, following the application of a commercial analog formulation containing Pal-GHK at quantities of <100 ppm (see *SI Appendix* for Franz cell method details). The data presented in Fig. 4*A* demonstrate that the Pal-GHK molecular ion [M+H]^+^ decreases in intensity with increasing skin depth, reaching a baseline level at the SC–epidermal boundary and hence showing delivery within the SC layer.

We were also able to monitor the permeation of this compound through individual layers of the SC, through an analysis of tape strip samples collected from human volunteers in vivo, following the application of a commercial analog formulation containing Pal-GHK (method details in *SI Appendix*). These data, presented in Fig. 4*B*, support the findings from the ex vivo depth profile method, confirming that this Pal-GHK molecule is present at significant levels above the baseline level throughout the SC.

Kawashima et al. (53) have recently demonstrated the analysis of a similar collagen tripeptide in skin using ToF-SIMS. However, despite using a highly concentrated solution (10% by weight), their results state that the molecular ion peak of the tripeptide was “scarcely detected” in the second layer of the SC and a multivariate analysis was needed to confirm its presence. Our analysis was conducted following application of a complex formulation with the active compound at levels of <100 ppm, so 1,000-fold less. Yet, despite its low concentration within the skin and its chemical similarity to native skin components, we were able to clearly identify the molecular [M+H^+^] ion (C_30_H_55_N_6_O_5_^+^) and assess its permeation as a function of skin depth from human in vitro and in vivo delivery.

Overall, our study has illustrated that the 3D OrbiSIMS technique, both single-beam depth profiling and 2D high resolution imaging, can provide a detailed molecular characterization of the SC, by investigating the intricate distribution of both native and exogenous species within this tissue. The data presented have demonstrated the huge potential of this approach to provide molecular information for skin research, aiding the understanding of both fundamental biological processes and pharmaceutical delivery.

## Materials and Methods

### Skin Acquisition.

In our study, ex vivo porcine skin refers to full-thickness skin taken from the posterior ear tissue of 6-mo-old pigs (full method in *SI Appendix*) and ex vivo human skin refers to full-thickness skin tissue that was purchased from Tissue Solutions (https://www.tissue-solutions.com/). Skin tissue was obtained from three different Caucasian female donors, who were between 35 and 70 y old and all nonsmokers, and removed during cosmetic surgery. The specific details of each donor can be found in the supplementary information (*SI Appendix*, Table S9). The tissue was frozen immediately postsurgery, shipped, and stored at –20 °C prior to use.

Skin samples were also collected in vivo from the flexor forearms of one healthy volunteer, who participated after providing consent and confirming they were free from skin conditions and medication. In order to remove individual layers of the SC, tape-stripping experiments were performed using D-Squame skin sampling discs (CuDerm). The circular discs had a diameter of 22 mm and used a fully cured medical-grade synthetic polyacrylate ester adhesive. The disc was applied to the skin, a constant pressure was applied using a pressure disk applicator (CuDerm), and the disc was removed in one fluent motion. Consecutive tape strips were taken to remove individual corneocyte layers, with strip 1 specified as the uppermost layer of the SC. Samples were stored at −20 °C prior to analysis.

### 3D OrbiSIMS Single-Beam Depth Profile Analysis of Ex Vivo Skin.

For the analysis of frozen hydrated skin samples, the skin samples were first introduced into the airlock on the cryostage of the instrument. This was flooded with Argon gas and the cooling process initiated. Once the stage had reached −80 °C, the vacuum pump was initiated, which accelerated the cooling process and brought the stage, and consequently skin, temperature down to −170 °C. During both the vacuum transfer and the analysis, the sample was maintained at this temperature using a closed-loop liquid nitrogen pumping system (IONTOF GmbH), which allowed stable cryooperation for more than 24 h, with a single dewar filling. The depth profiling settings were as follows: an Ar_3000_^+^ analysis beam with an energy of 20 keV and diameter of 20 µm was used as the primary ion beam. The duty cycle of the beam was set to 4.4% and the gas cluster ion beam (GCIB) current was 250 pA. The depth profile was run on an area of 200 × 200 µm using a sawtooth raster mode with crater size 280 × 280 µm. The cycle time was set to 200 μs. Optimal target potential varied for different samples, averaging at approximately ±384.5 V. Depth profiles were collected in both positive and negative polarity, in the mass range of 75 to 1,125 m/z and the injection time was set to 500 ms. Mass-resolving power was set to 240,000 at 200 m/z.

All data analysis was carried out using Surface Lab 7.1 (IONTOF GmbH). Using the software, some depth profiles have been compressed using a running average method (100 data points), where the individual data points represent the secondary ion intensities for a selected mass peak at subsequent depths of the sample. Where this has been done, it has been confirmed in the figure caption.

### 3D OrbiSIMS Analysis of Individual Corneocyte Layers.

#### Permeation analysis.

For permeation analysis, consecutive tape strips from healthy volunteers were used. A 3D OrbiSIMS analysis was performed on a hybrid instrument (IONTOF GmbH). For the acquisition of 3D OrbiSIMS permeation analysis, a 20 KeV Ar_3000_^+^ analysis beam with a diameter of 20 µm was used as the primary ion source. Samples were analyzed at RT across a 400 × 400 µm area in positive polarity with sawtooth raster mode and a crater size of 486 × 486 µm. Ar_3000_^+^ primary ions were used with a target current of 230 pA with charge compensation performed using a low-energy (20 eV) electron flood gun. Duty cycle was set to 4.4% and cycle time to 200 µs. Argon gas flooding was in operation to aid with charge compensation, which led to a pressure of 9.0 × 10^−7^ bar in the main chamber. The optimal target potential was at approximately +130 V at average. Mass spectra were recorded at a resolution of 240,000 at m/z 200 in the mass range of 75 to 1,125 m/z. The automatic gain control (AGC) target was off with the maximum injection time set at 500 ms. A total of 30 scans were conducted for each tape strip as well as 3 areas per tape strip as technical replicates. Both data acquisition and the subsequent data processing were performed using SurfaceLab 7 software (IONTOF GmbH). Orbitrap data were acquired using a Thermo Fisher Orbitrap HF mass spectrometer. Assignments were determined by accurate mass within 3 ppm error of the calculated mass.

#### High-resolution imaging.

The images were acquired using a 20 KeV Ar_1700_^+^ cluster ion beam with a primary ion current of 9 pA. The beam size was around 2 µm in diameter, but the final pixel size was set to 4 µm to reduce image acquisition time. The primary ion dose was distributed equally across each pixel area by rastering the beam in a 10 × 10 pixel microraster. Four imaging scans were acquired from a field of view area of 500 × 500 µm^2^ leading to a total primary ion dose density of 3.53× 10^14^ ions/cm^2^. Charge compensation was performed by flooding the sample using 20 eV electrons. The mass resolution was set to 240,000 (at m/z 200) and a fixed injection time of 500 ms was used.

### SIMS-MFP Software.

To help perform a more comprehensive search on classes of compounds present in the data, SIMS-MFP software was used. This software is optimized for SIMS data and is based on molecular formula matching, first described by Kind et al. (54). This was used to perform molecular formula prediction over the negative ion depth profile 3D OrbiSIMS dataset acquired from frozen hydrated human skin. After performing a peak search using the SurfaceLab software (IONTOF, GmbH) (minimum count = 70,000), we created a dataset containing 11,000 discrete ion signals. We searched for compounds containing C_n_H_n_O_2_^−^ (fatty acids), C_n_H_n_O_6_^−^ (triglycerides), C_n_H_n_O_n_N_1_^−^ (ceramides), C_n_H_n_O_n_S_1_^−^ (sulfates), C_<6_H_<20_N_<4_O_<3_^−^ (amino acids), and C_n_H_n_O_n_N_n_P_1_^−^ (phospholipids) where *n* = any integer value and assignments were made from the output data tables. All assignments were made with <2 ppm mass deviation. The assignments were then verified by comparing against the LIPID MAPS Structure Database (42) and/or previous literature.

## Supplementary Material

Supplementary File

## Data Availability

Data can be found in the University of Nottingham data repository at https://doi.org/10.17639/nott.7170. All other data are included in the article and/or supporting information.
