# Supplementary material for: Elucidating the molecular landscape of the stratum corneum
Source: Proc Natl Acad Sci U S A. 2022 Mar 17;119(12):e2114380119. doi: 10.1073/pnas.2114380119 (PMC8944899; doi:10.1073/pnas.2114380119)
Supplement: Supplementary File [file pnas.2114380119.sapp.pdf]

PNAS
www.pnas.org

Supplementary Information for

Elucidating the molecular landscape of the *stratum corneum*

Nichola J. Starr [a], Mohammed H. Khan [a], Max K. Edney [b], Gustavo F. Trindade [a, c], Stefanie Kern [a] Alexander Pirkl [d], Matthias Kleine-Boymann [d], Christopher Elms [e], Mark M. O'Mahony [e], Mike Bell [e], Morgan R. Alexander [a] and David J. Scurr [a*]

[a] School of Pharmacy, University of Nottingham, University Park, Nottingham, NG7 2RD, UK

[b] Department of Chemical and Environmental Engineering, University of Nottingham, University Park, Nottingham, NG7 2RD, UK

[c] National Centre of Excellence in Mass Spectrometry Imaging, National Physical Laboratory, Hampton Road, Teddington, Middlesex, TW11 0LW, UK

[d] Research and Development, IONTOF GmbH, 48149 Münster, Germany

[e] No7 Beauty Company, Walgreens Boots Alliance, Thane Road, Nottingham, NG90 1BS, UK

*Corresponding author: David J. Scurr

Email: david.scurr@nottingham.ac.uk

This PDF file includes:

Supplementary text
Figures S1 to S11 (not allowed for Brief Reports)
Tables S1 to S9 (not allowed for Brief Reports)
SI References

Supplementary Information Text

Materials and Methods

Porcine skin acquisition. Porcine skin was obtained post-slaughter from pigs reared specifically for food, which ensured that removing the skin tissue was a secondary use of the animal. The ears were removed immediately after slaughter, prior to cleaning or hair removal in order to maintain skin integrity. The skin was stored for a maximum of 24 h at 4 °C, after which the tissue was washed with ultrapure water only and the hair trimmed with scissors. Using a scalpel, the outer-ear skin was detached from the underlying cartilage and any excess subcutaneous fat was removed. The skin was wrapped in aluminum foil and stored at −20 °C for a maximum of 3 months prior to use. Full thickness skin sections of approximately 2 mm were used for analysis.

In vitro **Permeation Studies.** Once defrosted, circular *ex vivo* human skin was cut and mounted, dermal side down, between a donor and receptor chamber in a Franz-type static diffusion cell set-up (1) with an exposed surface area of 1.1 cm^2 and a receptor compartment volume of 3 mL. The receptor compartment was filled with Phosphate Buffered Saline (pH 7.5) and an infinite dose (200 mg) of a commercial analogue formulation containing Pal-GHK at <100 ppm was applied via the donor compartment. Sink conditions were maintained throughout the experiments. The skin was exposed to the formulation for 4 h in a water bath set to 36.5 °C, which maintains a skin temperature of 32°C. After 4 h, the Franz cell was dismantled and the excess formulation was removed with a dry sponge. The skin was wiped with a sponge soaked in Teepol Solution (3 % v/v) and then dehydrated under vacuum at RT for 24 h before loading into the Hybrid-SIMS instrument for analysis. As the focus of the analysis was an exogenous peptide and not a native compound, for practical and time saving reasons we chose to prepare the samples under dehydrated conditions rather than frozen hydrated.

In vivo **Permeation Studies.** Three Caucasian subjects (2 females, 1 male) aged between 30-55 were used for the study. All subjects gave informed consent to participate. The study was performed in a constant environment of 22 ± 2 °C and 50 ± 5 % humidity and subjects were acclimatized for 15 minutes prior to product application. A commercial analogue formulation containing Pal-GHK at <100 ppm was applied to one site on the volar forearm at 2 mg/cm^2 and one site was left untreated. Four hours post-application the sites were gently wiped with a tissue and 15 sequential tape strips were collected from each site using D-squame® strips (CuDerm). A D-squame® pressure instrument (CuDerm) was used to apply consistent pressure for 5 seconds before removing strips from the skin. Tape strips were then stored at 4°C until analysis.

Supplementary Information Figures

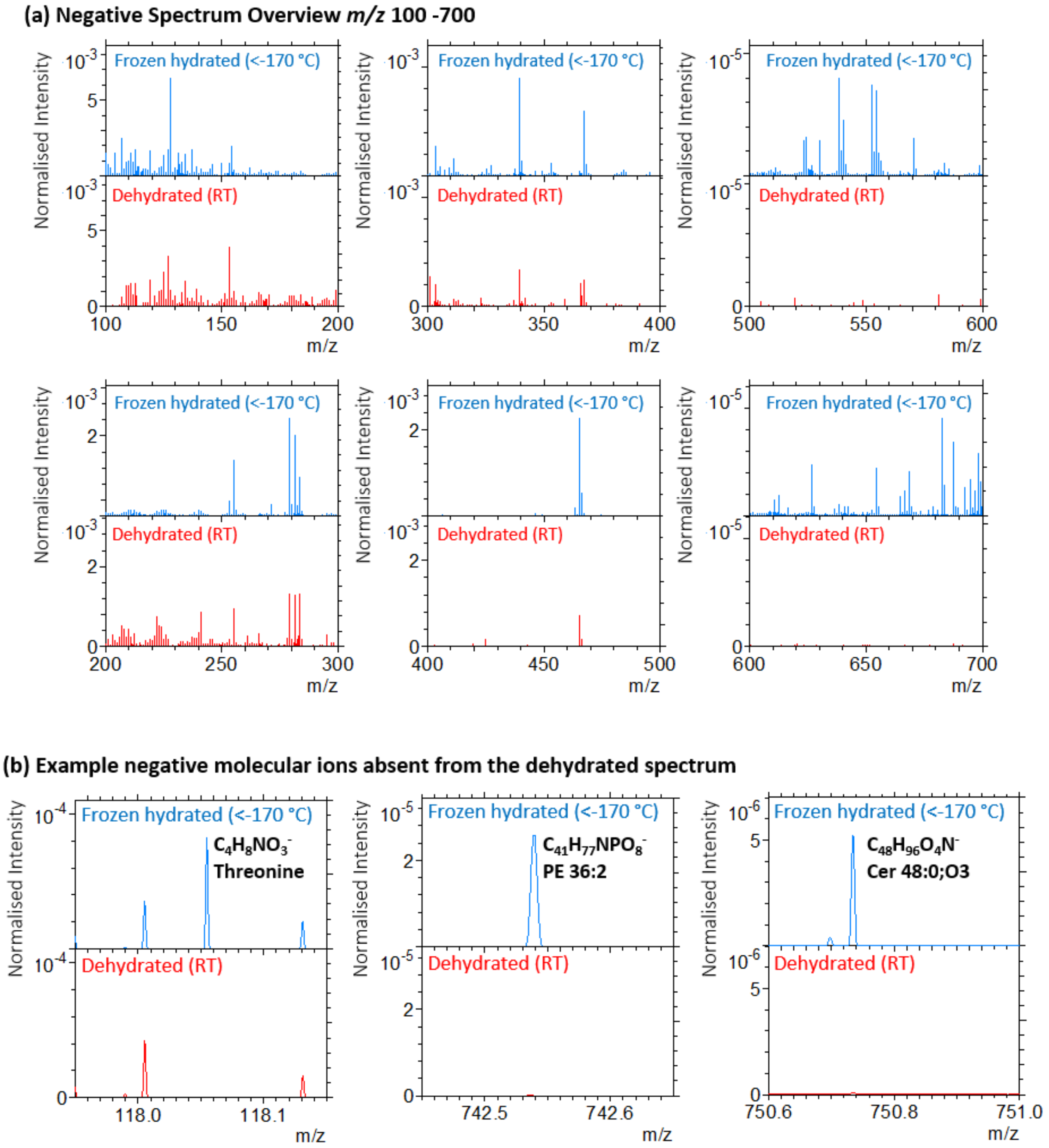

Figure S1. OrbiSIMS negative polarity spectral data, showing a comparison of the spectra for frozen hydrated (<-170 °C) vs. dehydrated (RT) *ex vivo* porcine skin tissue. The primary ion beam was Ar_{3000}^{+}. The total applied ion dose for the frozen hydrated and dehydrated sample was 1.8 x 10^{13} and 6.79 x 10^{13} respectively. **(a)** An overview of the spectrum from *m/z* 100 -700. The ion intensity has been normalized to the total ion count. **(b)** Three example ion peaks that are absent from the dehydrated spectrum. Specifically, the putatively assigned molecular ions of amino acid threonine ($C_4H_8NO_3^-$), phospholipid PE 36:2 ($C_{41}H_{77}NPO_8^-$) and ceramide C48:0;O3 ($C_{48}H_{96}O_4N^-$).

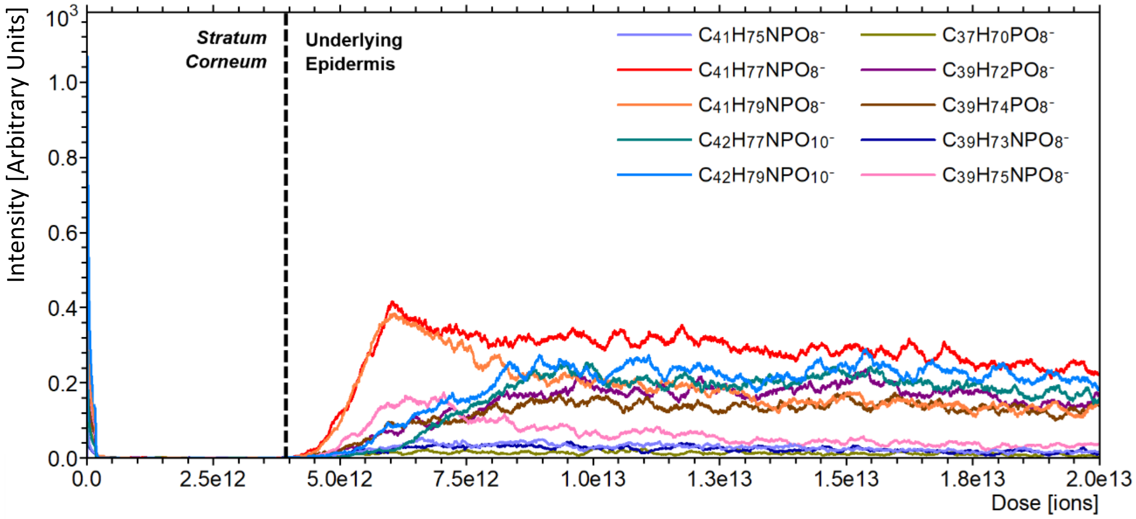

Figure S2. OrbiSIMS negative polarity depth profile data showing the ion intensity variation as function of ion dose/skin depth for the putatively assigned phospholipid species. The primary ion beam was Ar_{3000}^{+}. The 10 most intense ions are presented. These ions have been used to determine the *stratum corneum* – epidermal boundary, which is approximated with a dotted vertical line. The profile has been compressed using a running average method (100 data points).

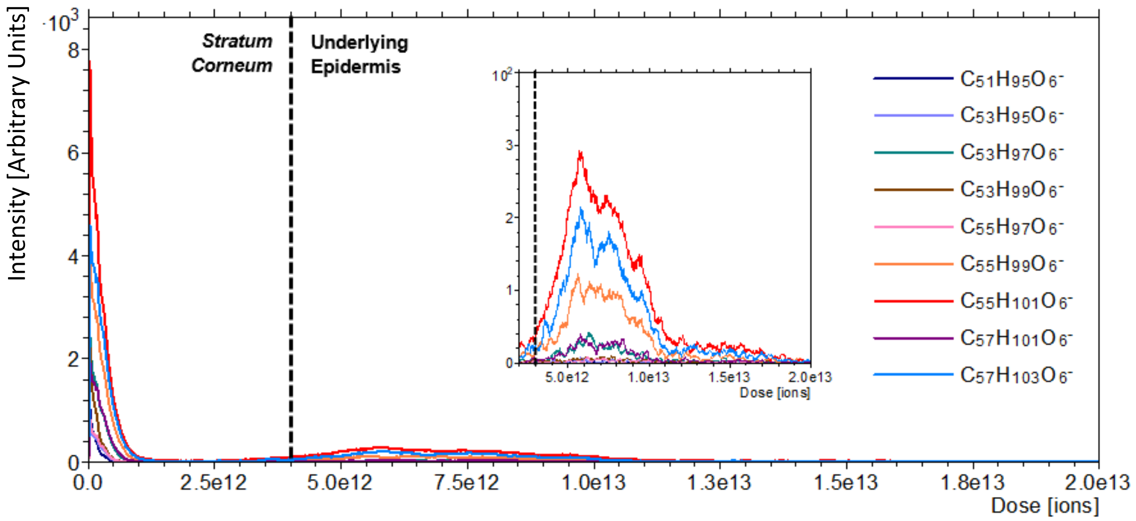

Figure S3. OrbiSIMS negative polarity depth profile data showing the ion intensity variation as function of ion dose/skin depth for the putatively assigned triglyceride species. The inset is a zoom in of the region between 2 x 10^{12} and 2 x 10^{13} ion dose. The primary ion beam was Ar_{3000}^{+}. The *stratum corneum* – epidermal boundary has been determined, and approximated with a dotted vertical line, based on the ion intensity variation of the phospholipid species (Figure S2). The profile has been compressed using a running average method (100 data points).

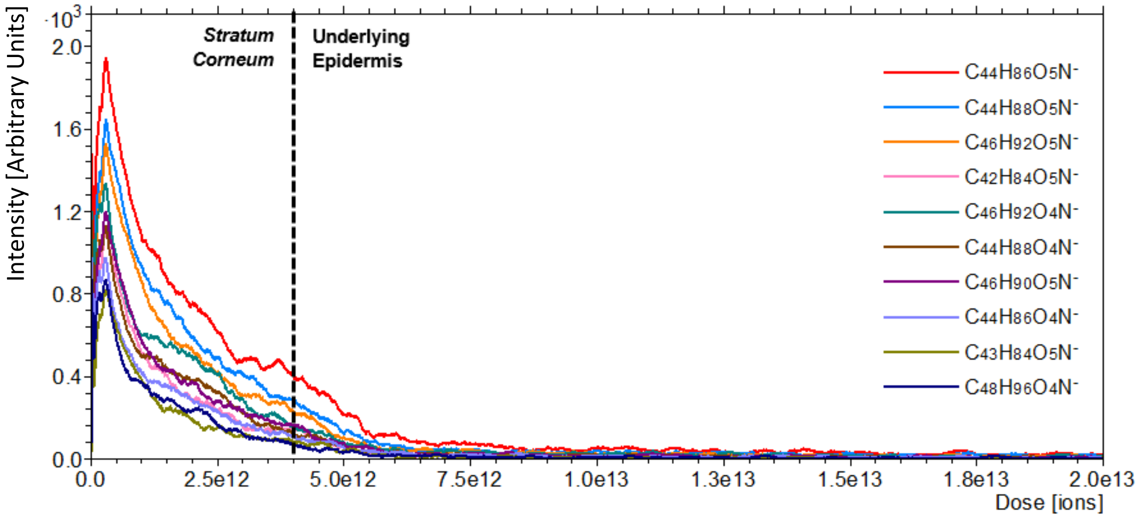

Figure S4. OrbiSIMS negative polarity depth profile data showing the ion intensity variation as function of ion dose/skin depth for the putatively assigned ceramide species. The primary ion beam was Ar_{3000}^+. The *stratum corneum* – epidermal boundary has been determined, and approximated with a dotted vertical line, based on the ion intensity variation of the phospholipid species (Figure S2). The profile has been compressed using a running average method (100 data points).

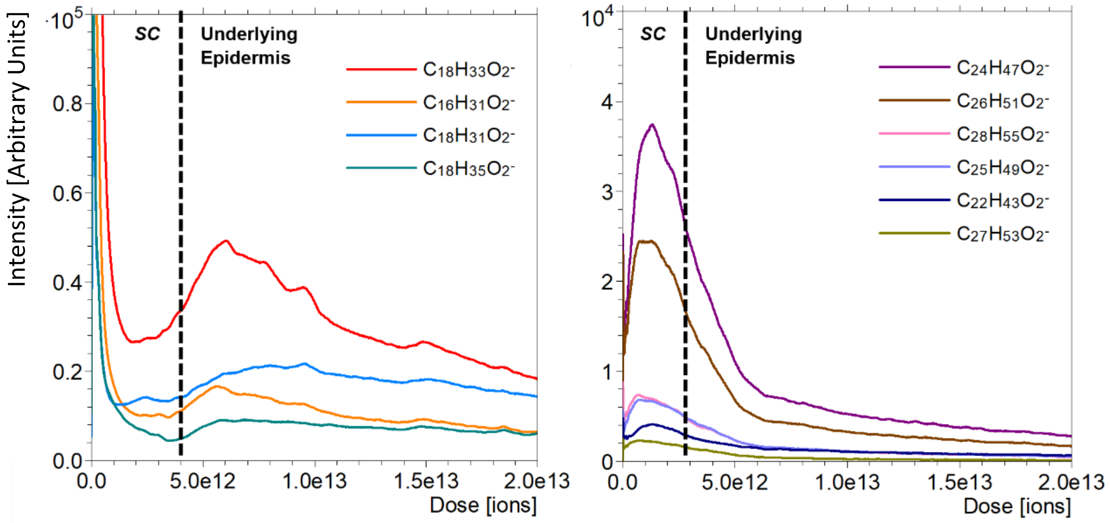

Figure S5. OrbiSIMS negative polarity depth profile data showing the ion intensity variation as function of ion dose/skin depth for the putatively assigned fatty acid species. This class of compounds presented two different trends with depth. The primary ion beam was Ar_{3000}^{+}. The *stratum corneum* – epidermal boundary has been determined, and approximated with a dotted vertical line, based on the ion intensity variation of the phospholipid species (Figure S2). The profile has been compressed using a running average method (100 data points).

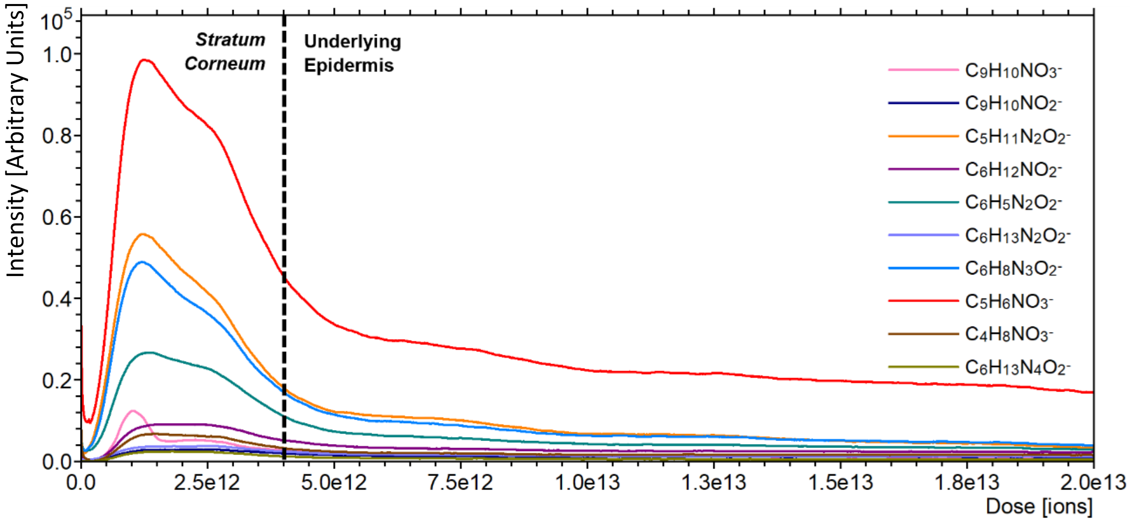

Figure S6. 3D OrbiSIMS negative polarity depth profile data showing the ion intensity variation as function of ion dose/skin depth for the putatively assigned amino acid species. The primary ion beam was Ar_{3000}^{+}. The *stratum corneum* – epidermal boundary has been determined, and approximated with a dotted vertical line, based on the ion intensity variation of the phospholipid species (Figure S2). The profile has been compressed using a running average method (100 data points).

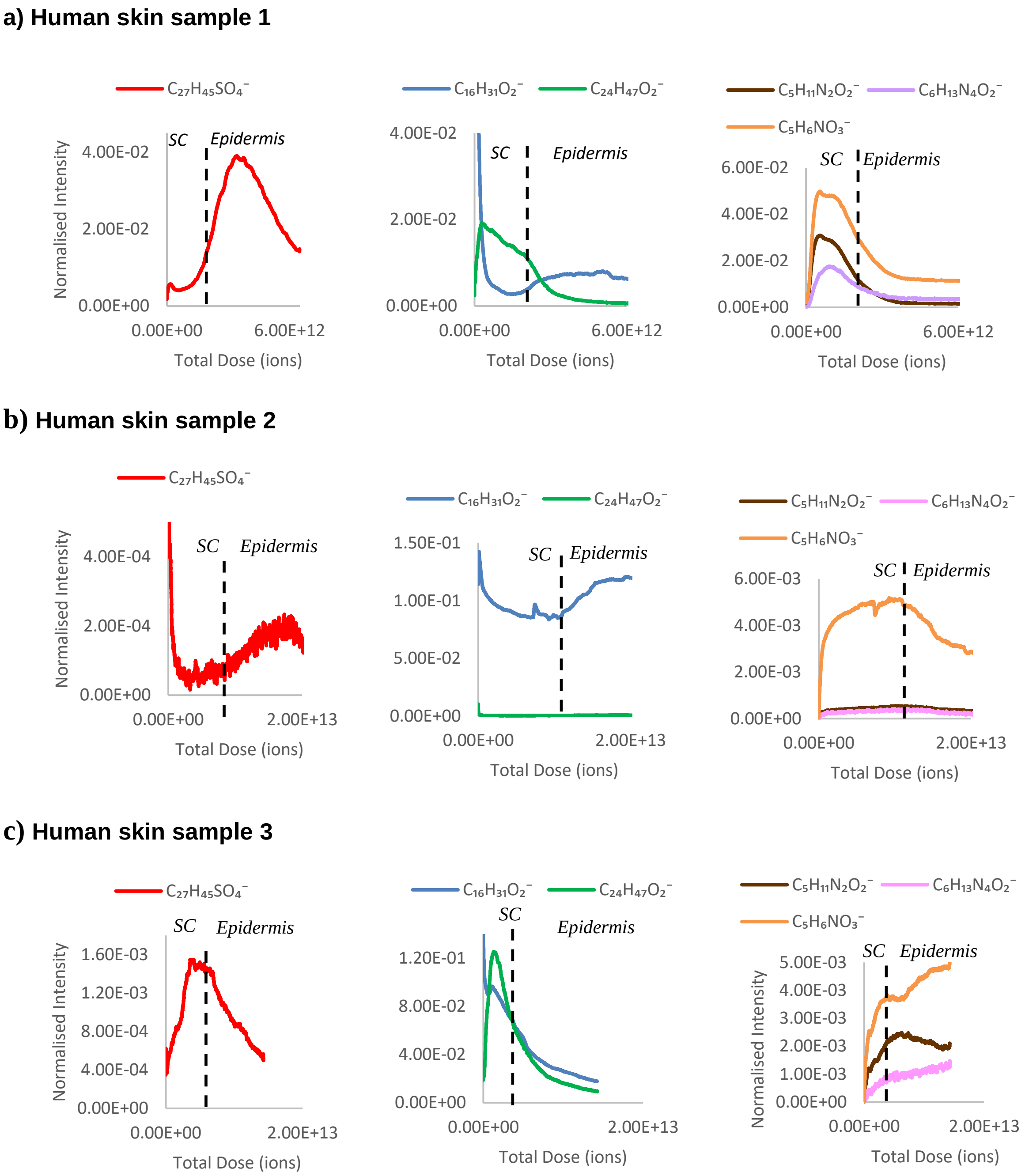

Figure S7: OrbiSIMS data showing the ion intensity variation as a function of skin depth for various native compounds in three different human skin samples (SI Table S6) with sample 1 analyzed in the cryo hydrated state and samples 2 and 3 analyzed in a dehydrated state. The ion intensities have been normalized to the total ion count. The variation of depth distributions of the following compounds was assessed; cholesterol sulfate ($C_{27}H_{45}SO_4^-$), palmitic acid ($C_{16}H_{31}O_2^-$), lignoceric acid ($C_{24}H_{47}O_2^-$), arginine ($C_6H_{13}N_4O_2^-$), PCA ($C_5H_6NO_3^-$) and ornithine ($C_5H_{11}N_2O_2^-$).

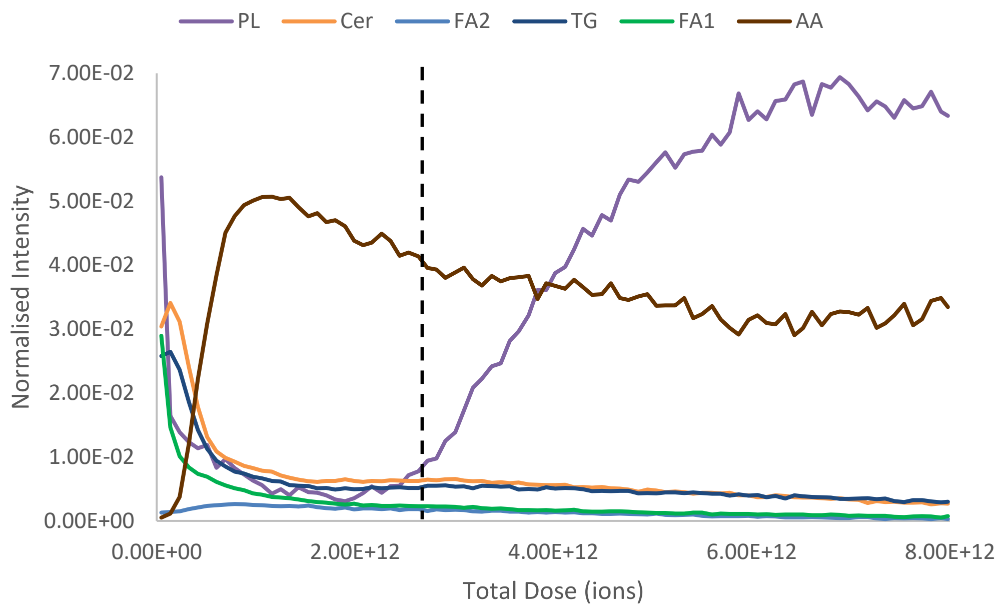

Figure S8: OrbiSIMS positive polarity depth profile data showing the ion intensity variation as function of ion dose/skin depth for various putatively assigned compounds. The primary ion beam was Ar_{3000}^{+}. The ion intensities have been normalized to the total ion count and the profiles have been compressed using a running average method (using a 100 data point average). The *stratum corneum* – epidermal boundary, approximated with a dotted vertical line, has been determined based on the ion intensity variation of the phospholipid species (Figure S2). Example ions from each identified compound class: PL – phospholipids ($C_5H_{15}NPO_4^{+}$), TG – triglycerides ($C_7H_{11}^{+}$), Cer – ceramides ($C_{39}H_{71}O_4^{+}$), FA1 and FA2 – fatty acids ($C_{18}H_{35}O_2^{+}$ and $C_{26}H_{56}NO_2^{+}$) and AA – amino acids ($C_6H_{15}N_4O_2^{+}$). The normalized intensity for the phospholipid and amino acid ions have been multiplied by a factor of 10.

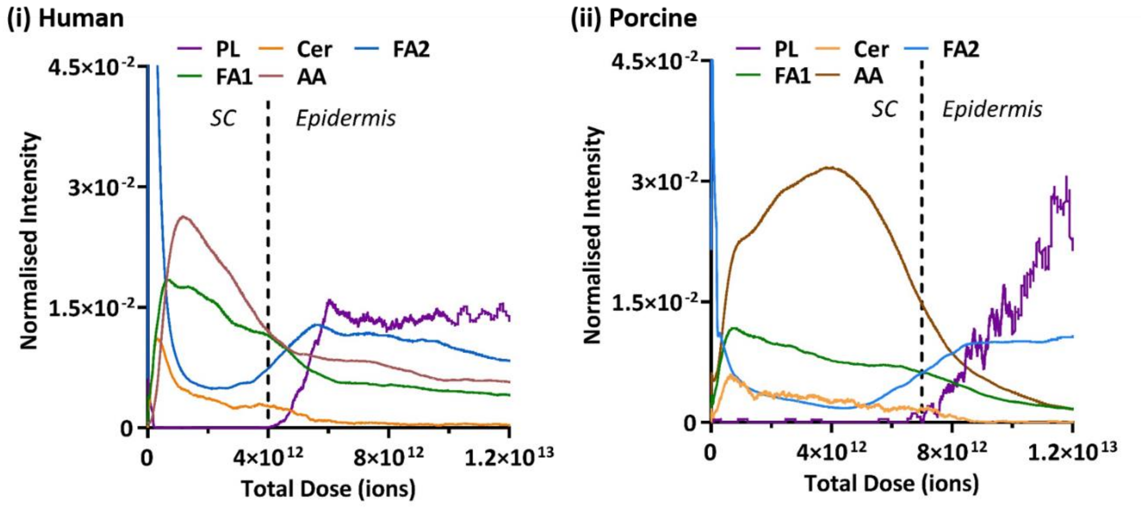

Figure S9. Data produced from 3D OrbiSIMS negative polarity depth profile analysis of frozen hydrated *ex vivo* skin, both human and porcine. The primary ion beam was Ar_{3000}^{+}. Depth profiles for both (i) human and (ii) porcine samples, showing the ion intensity variation as function of ion dose/skin depth for various putatively assigned compounds. The ion intensities have been normalized to the total ion count and the profiles have been compressed using a running average method (using a 100 data point average). The *stratum corneum* – epidermal boundary, approximated with a dotted vertical line, has been determined for each species based on the ion intensity variation of the phospholipid ion. Example ions from the following compound classes are shown: PL – phospholipids ($C_{41}H_{77}NPO_8^-$), Cer – ceramides ($C_{44}H_{86}NO_5^-$), FA1 and FA2 – fatty acids ($C_{27}H_{47}O_2^-$ and $C_{16}H_{31}O_2^-$), AA – amino acids ($C_5H_{11}N_2O_2^-$). The normalized intensity for the porcine ceramide and phospholipid ions has been multiplied by a factor of 100 and 1000 respectively.

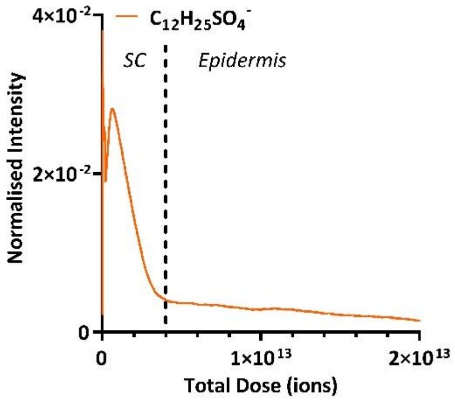

Figure S10. 3D OrbiSIMS negative polarity depth profile data showing the ion intensity variation as function of ion dose/skin depth for the putatively assigned exogenous compound sodium lauryl sulfate ($C_{12}H_{25}SO_4^-$). The primary ion beam was Ar_{3000}^+. The *stratum corneum* – epidermal boundary has been determined, and approximated with a dotted vertical line, based on the ion intensity variation of the phospholipid species (Figure S2). The ion intensity has been normalized to the total ion count and the profile has been compressed using a running average method (100 data points).

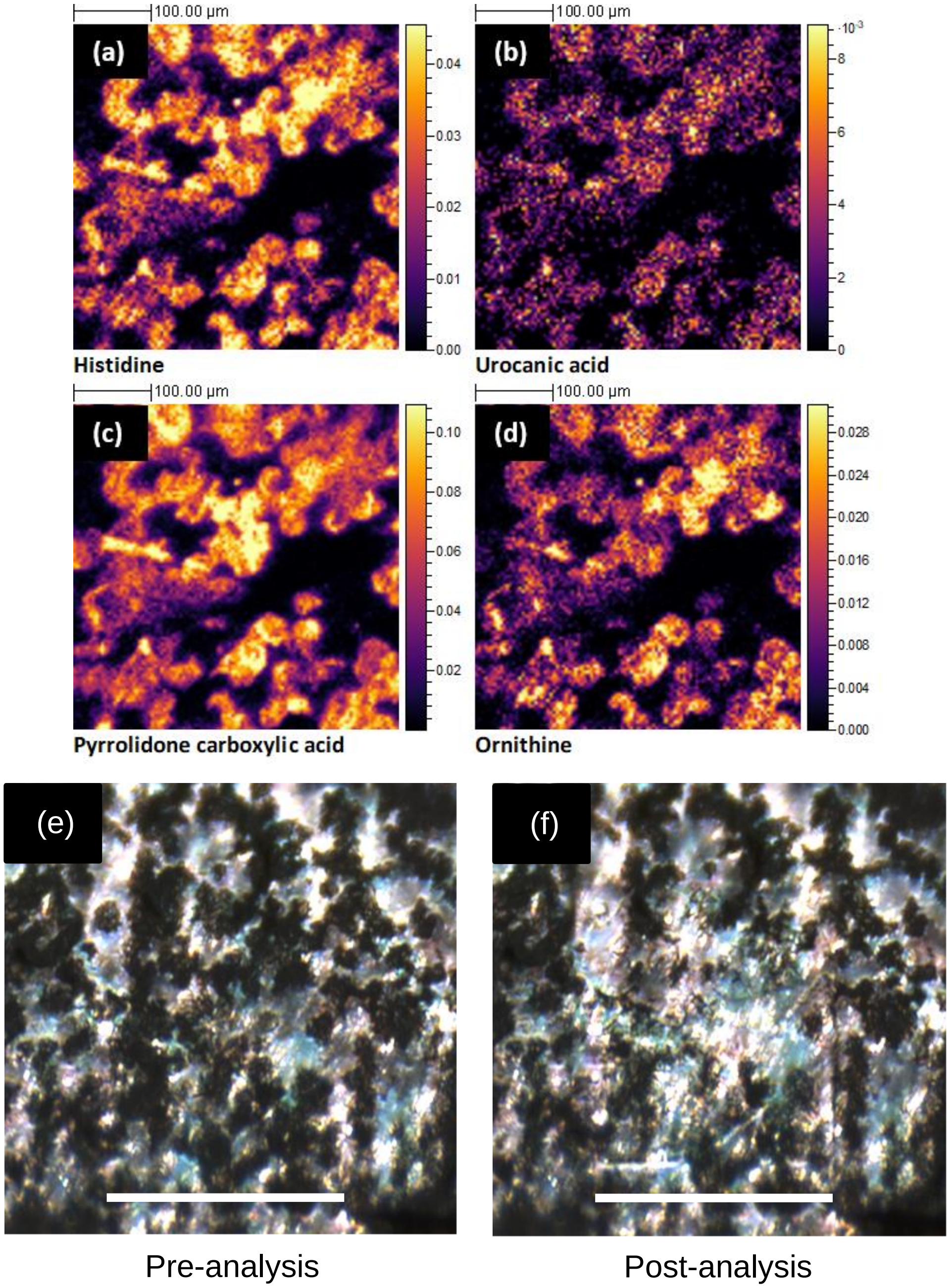

Figure S11. OrbiSIMS high resolution negative polarity ion images**,** produced from the analysis of an individual human corneocyte skin layer, collected *in vivo* using a tape stripping method. The data shown is from tape strip 7, which represents the middle of the *stratum corneum*. The ion images illustrate the 2D spatial distribution for the following molecular ions (a) Histidine ($C_6H_8N_3O_2^-$) (b) Urocanic acid ($C_6H_6N_2O_2^-$) (c) Pyrrolidone carboxylic acid ($C_5H_7NO_3^-$) (d) Ornithine ($C_5H_{12}N_2O_2^-$). All ion images have been normalized to the total ion image and their relative intensity scales set to 50 % of the maximum. Corresponding optical image taken pre and post analysis in (e) and (f) where white scale bar = 500 µm.

Supporting Information Tables

Table S1. Tabulated output from the SIMS-MFP software (2) showing the putative phospholipid assignments. The observed mass (*m/z*), chemical formula, molecular assignment and mass deviation are documented. All assignments were compared against the Lipid Maps Structural Database ® (3) and/or verified manually from previous literature.

Observed Mass (*m/z*)	Chemical Formula	Molecular Assignment	Mass Deviation
671.4656	$C_{37}H_{68}PO_8^-$	PA 34:2	0.7
673.4811	$C_{37}H_{70}PO_8^-$	PA 34:1	0.4
697.4811	$C_{39}H_{70}PO_8^-$	PA 36:3	0.4
699.4968	$C_{39}H_{72}PO_8^-$	PA 36:2	0.4
701.5125	$C_{39}H_{74}PO_8^-$	PA 36:1	-0.3
714.5076	$C_{39}H_{73}NPO_8^-$	PE 34:2	0.3
716.5234	$C_{39}H_{75}NPO_8^-$	PE 34:1	0.4
738.5076	$C_{41}H_{73}NPO_8^-$	PE 36:4	0.3
740.5234	$C_{41}H_{75}NPO_8^-$	PE 36:3	0.5
742.5391	$C_{41}H_{77}NPO_8^-$	PE 36:2	0.5
744.5548	$C_{41}H_{79}NPO_8^-$	PE 36:1	0.6
766.5390	$C_{43}H_{77}NPO_8^-$	PE 38:4	0.4
768.5543	$C_{43}H_{79}NPO_8^-$	PE 38:3	0.0
786.5287	$C_{42}H_{77}NPO_{10}^-$	PS 36:2	-0.4
788.5444	$C_{42}H_{79}NPO_{10}^-$	PS 36:1	0.4
812.5444	$C_{44}H_{79}NPO_{10}^-$	PS 38:3	0.3
835.5331	$C_{43}H_{80}PO_{13}^-$	PI 34:1	-0.7
861.5495	$C_{45}H_{82}PO_{13}^-$	PI 36:2	0.2
885.5499	$C_{47}H_{82}PO_{13}^-$	PI 38:4	0.7

Table S2. Tabulated output from the SIMS-MFP software (2) showing the putative triglyceride assignments. The observed mass (*m/z*), chemical formula, molecular assignment and mass deviation are documented. All assignments were compared against the Lipid Maps Structural Database ® (3) and/or verified manually from previous literature.

Observed Mass (*m/z*)	Chemical Formula	Molecular Assignment	Mass Deviation
803.7136	$C_{51}H_{95}O_6^-$	TG(16:0/14:0/18:1(9Z))	0.2
827.7136	$C_{53}H_{95}O_6^-$	TG(16:1(9Z)/16:1(9Z)/18:1(9Z))	0.2
829.7293	$C_{53}H_{97}O_6^-$	TG(16:1(9Z)/16:1(9Z)/18:0	0.0
831.7452	$C_{53}H_{99}O_6^-$	TG(16:0/16:1(9Z)/18:0)	0.2
853.7291	$C_{55}H_{97}O_6^-$	TG(18:2(9Z,12Z)/16:0/18:2	0.0
855.7449	$C_{55}H_{99}O_6^-$	TG(16:0/18:1(9Z)/18:2	-0.2
857.7606	$C_{55}H_{101}O_6^-$	TG(16:0/18:1/18:1)	-0.1
881.7605	$C_{57}H_{101}O_6^-$	TG(18:1/18:1/18:2)	-0.2
883.7762	$C_{57}H_{103}O_6^-$	TG(18:1(9E)/18:1(9E)/18:1	-0.1

Table S3. Tabulated output from the SIMS-MFP software (2) showing the putative ceramide assignments. The observed mass (*m/z*), chemical formula, molecular assignment and mass deviation are documented. All assignments were compared against the Lipid Maps Structural Database ® (3) and/or verified manually from previous literature.

Observed Mass (*m/z*)	Chemical Formula	Molecular Assignment	Mass Deviation
436.4160	$C_{28}H_{54}O_2N^-$	Cer 28:1;O	-0.5
438.4316	$C_{28}H_{56}O_2N^-$	Cer 28:0;O	-0.5
450.4317	$C_{29}H_{56}O_2N^-$	Cer 29:1;O	-0.7
452.4110	$C_{28}H_{54}O_3N^-$	Cer 28:1;O2	-0.1
454.4266	$C_{28}H_{56}O_3N^-$	Cer 28:0;O2	0
466.4265	$C_{29}H_{56}O_3N^-$	Cer 29:1;O2	-0.2
652.6253	$C_{41}H_{82}O_4N^-$	Cer 41:0;O3	0.2
654.6044	$C_{40}H_{80}O_5N^-$	Cer 40:0;O4	-0.1
666.6409	$C_{42}H_{84}O_4N^-$	Cer 42:0;O3	0.1
668.6202	$C_{41}H_{82}O_5N^-$	Cer 41:0;O4	0.2
678.6407	$C_{43}H_{84}O_4N^-$	Cer 43:1;O3	-0.2
680.6564	$C_{43}H_{86}O_4N^-$	Cer 43:0;O3	-0.2
680.6564	$C_{43}H_{86}O_5N^-$	Cer 43:0;O4	-0.1
682.6356	$C_{42}H_{84}O_5N^-$	Cer 42:0;O4	-0.2
692.6564	$C_{44}H_{86}O_4N^-$	ACer 44:0;O2 or Cer 44:1;O3*	-0.2
694.6357	$C_{43}H_{84}O_5N^-$	Cer 43:1;O4	-0.2
694.6721	$C_{44}H_{88}O_4N^-$	Cer 44:0;O3	-0.1
706.6721	$C_{45}H_{88}O_4N^-$	ACer 45:0;O2 or Cer 45:1;O3*	-0.1
708.6514	$C_{44}H_{86}O_5N^-$	ACer 44:0;O3 or Cer 44:1;O4*	-0.1
708.6877	$C_{45}H_{90}O_4N^-$	Cer 45:0;O3	-0.1
710.6671	$C_{44}H_{88}O_5N^-$	Cer 44:0;O4	-0.1
720.6878	$C_{46}H_{90}O_4N^-$	ACer 46:0;O2 or Cer 46:1;O3*	-0.1
722.6671	$C_{45}H_{88}O_5N^-$	ACer 45:0;O3 or Cer 45:1;O4*	-0.1
722.7035	$C_{46}H_{92}O_4N^-$	Cer 46:0;O3	0.0
724.6827	$C_{45}H_{90}O_5N^-$	Cer 45:0;O4	0.0
734.7034	$C_{47}H_{92}O_4N^-$	ACer 47:0;O2 or Cer 47:1;O3*	0.0
736.6827	$C_{46}H_{90}O_5N^-$	ACer 46:0;O3 or Cer 46:1;O4*	0.0
736.7192	$C_{47}H_{94}O_4N^-$	Cer 47:0;O3	0.0
738.6985	$C_{46}H_{92}O_5N^-$	Cer 46:0;O4	0.0
748.7189	$C_{48}H_{94}O_4N^-$	ACer 48:0;O2 or Cer 48:1;O3*	-0.4
750.6981	$C_{47}H_{92}O_5N^-$	ACer 47:0;O3 or Cer 47:1;O4*	0.0
750.7346	$C_{48}H_{96}O_4N^-$	Cer 48:0;O3	-0.3
752.7138	$C_{47}H_{94}O_5N^-$	Cer 47:0;O4	-0.3
764.7139	$C_{48}H_{94}O_5N^-$	ACer 48:0;O3 or Cer 48:1;O4*	0.0
764.7502	$C_{49}H_{98}O_4N^-$	Cer 49:0;O3	-0.1
766.7295	$C_{48}H_{96}O_5N^-$	Cer 48:0;O4	-0.2
778.7659	$C_{50}H_{100}O_4N^-$	Cer 50:0;O3	-0.1

* Without further investigation we were unable to distinguish whether the structure related to the ceramide or the acyl-ceramide.

Table S4. Tabulated output from the SIMS-MFP software (2) showing the putative fatty acid assignments. The observed mass (*m/z*), chemical formula, molecular assignment and mass deviation are documented. All assignments were compared against the Lipid Maps Structural Database ® (3) and/or verified manually from previous literature.

Observed Mass (*m/z*)	Chemical Formula	Molecular Assignment	Mass Deviation
235.1704	$C_{15}H_{23}O_{2-}$	FA 15:3	0
237.1861	$C_{15}H_{25}O_{2-}$	FA 15:2	0
239.2017	$C_{15}H_{29}O_{2-}$	FA 15:1	0.1
241.2174	$C_{15}H_{29}O_{2-}$	FA 15:0	0.1
249.1861	$C_{16}H_{25}O_{2-}$	FA 16:3	0.3
251.2018	$C_{16}H_{27}O_{2-}$	FA 16:2	0.3
255.2332	$C_{16}H_{31}O_{2-}$	FA 16:0	0.5
263.2019	$C_{17}H_{27}O_{2-}$	FA 17:3	0.5
265.2175	$C_{17}H_{29}O_{2-}$	FA 17:2	0.6
267.2332	$C_{17}H_{31}O_{2-}$	FA 17:1	0.6
269.2489	$C_{17}H_{33}O_{2-}$	FA 17:0	0.7
277.2174	$C_{18}H_{29}O_{2-}$	FA 18:3	0
279.2331	$C_{18}H_{31}O_{2-}$	FA 18:2	0.1
281.2488	$C_{18}H_{33}O_{2-}$	FA 18:1	0.2
283.2644	$C_{18}H_{35}O_{2-}$	FA 18:0	0.1
291.2332	$C_{19}H_{31}O_{2-}$	FA 19:3	0.4
293.2488	$C_{19}H_{33}O_{2-}$	FA 19:2	-0.5
295.2642	$C_{19}H_{35}O_{2-}$	FA 19:1	-0.5
297.2799	$C_{19}H_{37}O_{2-}$	FA 19:0	-0.4
303.233	$C_{20}H_{31}O_{2-}$	FA 20:4	-0.3
305.2487	$C_{20}H_{33}O_{2-}$	FA 20:3	-0.2
307.2643	$C_{20}H_{35}O_{2-}$	FA 20:2	-0.1
309.2799	$C_{20}H_{37}O_{2-}$	FA 20:1	-0.1
311.2957	$C_{20}H_{39}O_{2-}$	FA 20:0	0
319.2641	$C_{21}H_{35}O_{2-}$	FA 21:3	-0.8
321.2798	$C_{21}H_{37}O_{2-}$	FA 21:2	-0.7
323.2955	$C_{21}H_{39}O_{2-}$	FA 21:1	-0.2
325.3111	$C_{21}H_{41}O_{2-}$	FA 21:0	-0.6
331.2642	$C_{22}H_{35}O_{2-}$	FA 22:4	-0.4
333.2799	$C_{22}H_{37}O_{2-}$	FA 22:3	-0.2
335.2956	$C_{22}H_{39}O_{2-}$	FA 22:2	-0.2
337.3113	$C_{22}H_{41}O_{2-}$	FA 22:1	-0.2
339.3267	$C_{22}H_{43}O_{2-}$	FA 22:0	-0.9
349.3111	$C_{23}H_{41}O_{2-}$	FA 23:2	-0.5
351.3268	$C_{23}H_{43}O_{2-}$	FA 23:1	-0.5
353.3424	$C_{23}H_{45}O_{2-}$	FA 23:0	-0.5
365.3423	$C_{24}H_{45}O_{2-}$	FA 24:1	-0.9
367.3581	$C_{24}H_{47}O_{2-}$	FA 24:0	-0.8
377.3424	$C_{25}H_{45}O_{2-}$	FA 25:2	-0.5
379.3581	$C_{25}H_{47}O_{2-}$	FA 25:1	-0.5
381.3737	$C_{25}H_{49}O_{2-}$	FA 25:0	-0.6
391.3583	$C_{26}H_{47}O_{2-}$	FA 26:2	-0.1
393.3739	$C_{26}H_{49}O_{2-}$	FA 26:1	-0.1
395.3894	$C_{26}H_{51}O_{2-}$	FA 26:0	-0.8
405.3738	$C_{27}H_{49}O_{2-}$	FA 27:2	-0.4

407.3894	$C_{27}H_{51}O_{2-}$	FA 27:1	-0.4
409.4051	$C_{27}H_{53}O_{2-}$	FA 27:0	-0.5
419.3895	$C_{28}H_{51}O_{2-}$	FA 28:2	0
421.4052	$C_{28}H_{53}O_{2-}$	FA 28:1	-0.2
423.4208	$C_{28}H_{55}O_{2-}$	FA 28:0	-0.2
433.405	$C_{29}H_{53}O_{2-}$	FA 29:2	-0.3
435.4207	$C_{29}H_{55}O_{2-}$	FA 29:1	-0.5
437.4364	$C_{29}H_{57}O_{2-}$	FA 29:0	-0.5
447.4208	$C_{30}H_{55}O_{2-}$	FA 30:2	-0.1
449.4365	$C_{30}H_{57}O_{2-}$	FA 30:1	-0.3
451.4521	$C_{30}H_{59}O_{2-}$	FA 30:0	-0.3
463.4522	$C_{31}H_{59}O_{2-}$	FA 31:1	0
465.4676	$C_{31}H_{61}O_{2-}$	FA 31:0	-0.6
475.452	$C_{32}H_{59}O_{2-}$	FA 32:2	-0.6
477.4676	$C_{32}H_{61}O_{2-}$	FA 32:1	-0.5
479.4833	$C_{32}H_{63}O_{2-}$	FA 32:0	-0.5

Table S5. Tabulated output from the SIMS-MFP software (2) showing the putative amino acid assignments. The observed mass (*m/z*), chemical formula, molecular assignment and mass deviation are documented. All assignments were compared against the Lipid Maps Structural Database ® (3) and/or verified manually from previous literature.

Observed Mass (*m/z*)	Chemical Formula	Molecular Assignment	Mass Deviation
118.0509	$C_4H_8NO_3^-$	Threonine	-0.3
128.0353	$C_5H_6NO_3^-$	Pyroglutamic acid	-0.5
130.0872	$C_6H_{12}NO_2^-$	Leucine	-1.0
131.0825	$C_5H_{11}N_2O_2^-$	Ornithine	-0.8
137.0355	$C_6H_5N_2O_2^-$	Urocanic acid	-0.9
145.0981	$C_6H_{13}N_2O_2^-$	Lysine	-1.0
154.0621	$C_6H_8N_3O_2^-$	Histidine	-0.6
164.0716	$C_9H_{10}NO_2^-$	Phenylalanine	-0.6
173.1042	$C_6H_{13}N_4O_2^-$	Arginine	-1.0
180.0665	$C_9H_{10}NO_3^-$	Tyrosine	-0.6

Table S6. Tabulated output from the SIMS-MFP software (2) showing the putative ceramide assignments in positive mode. The observed mass (*m/z*), chemical formula, molecular assignment and mass deviation are documented. All assignments were compared against the Lipid Maps Structural Database ® (3) and/or verified manually from previous literature.

Observed Mass (*m/z*)	Chemical Formula	Molecular Assignment	Mass Deviation
578.5131	$C_{36}H_{68}O_4N_+$	Cer 36:3;O3	2.0
638.6079	$C_{40}H_{80}O_4N_+$	Cer 40:1;O3	-0.4
666.6390	$C_{42}H_{84}O_5N_+$	Cer 42:1;O4	-0.7

Table S7. Tabulated output from the SIMS-MFP software (2) showing the putative fatty acid assignments in positive mode. The observed mass (*m/z*), chemical formula, molecular assignment and mass deviation are documented. All assignments were compared against the Lipid Maps Structural Database ® (3) and/or verified manually from previous literature.

Observed Mass (*m/z*)	Chemical Formula	Molecular Assignment	Mass Deviation
101.0597	$C_5H_9O_{2+}$	FA 5:1	0.0
115.0752	$C_6H_{11}O_{2+}$	FA 6:1	1.5
115.0752	$C_6H_{11}O_{2+}$	FA 6:1	1.5
255.2316	$C_{16}H_{31}O_{2+}$	FA 16:1	-1.0
257.2473	$C_{16}H_{33}O_{2+}$	FA 16:0	-0.8
281.2473	$C_{18}H_{33}O_{2+}$	FA 18:2	-0.7
283.2630	$C_{18}H_{35}O_{2+}$	FA 18:1	-0.5
297.2786	$C_{19}H_{37}O_{2+}$	FA 19:1	0.6
307.2630	$C_{20}H_{35}O_{2+}$	FA 20:3	-0.5
309.2786	$C_{20}H_{37}O_{2+}$	FA 20:2	-0.6
311.2942	$C_{20}H_{39}O_{2+}$	FA 20:1	-0.8
323.2943	$C_{21}H_{39}O_{2+}$	FA 21:2	0.4

Table S8. Tabulated output from the SIMS-MFP software (2) showing the putative amino acid assignments in positive mode. The observed mass (*m/z*), chemical formula, molecular assignment and mass deviation are documented. All assignments were compared against the Lipid Maps Structural Database ® (3) and/or verified manually from previous literature.

Observed Mass (*m/z*)	Chemical Formula	Molecular Assignment	Mass Deviation
120.0654	$C_4H_{10}NO_3^+$	Threonine	-1.0
130.0497	$C_5H_8NO_3^+$	Pyroglutamic acid	-1.3
132.1017	$C_6H_{14}NO_2^+$	Leucine	-1.6
133.0970	$C_5H_{13}N_2O_2^+$	Ornithine	-1.2
139.0500	$C_6H_7N_2O_2^+$	Urocanic acid	-1.2
147.1126	$C_6H_{15}N_2O_2^+$	Lysine	-1.4
156.0765	$C_6H_{10}N_3O_2^+$	Histidine	-1.6
166.0860	$C_9H_{12}NO_2^+$	Phenylalanine	-1.5
175.1187	$C_6H_{15}N_4O_2^+$	Arginine	-1.4
182.0810	$C_9H_{12}NO_3^+$	Tyrosine	-0.9

Table S9. *Ex vivo* Human Skin Donor Information.

Donor #	Age	Location	Ethnicity
1	38	Abdomen	Caucasian
2	53	Arm	Caucasian
3	69	Abdomen	Caucasian

SI References

1. Franz TJ (1975) Percutaneous absorption on the relevance of in vitro data. The Journal of investigative dermatology 64(3):190-195.
2. Sud M*, et al.* (2007) LMSD: LIPID MAPS structure database. *Nucleic acids research* 35(Database issue):D527-532
3. Edney MK, *et al.* (2021) Molecular Formula Prediction for Chemical Filtering of 3D OrbiSIMS Datasets. *Analytical Chemistry* https://doi.org/10.1021/acs.analchem.1c04898.
